# Equitable access to COVID-19 vaccines in Botswana: a scoping review

**DOI:** 10.3389/frhs.2025.1609089

**Published:** 2025-07-29

**Authors:** John T. Tlhakanelo, John Ele-Ojo Ataguba, Vincent Pagiwa, Nankie Ramabu, Khutsafalo Kadimo, Dintle Molosiwa, Grace Njeri Muriithi, Daniel Malik Achala, Elizabeth Naa Adukwei Adote, Chinyere Ojiugo Mbachu, Senait Alemayehu Beshah, Nyasha Masuka, Chijioke Osinachi Nwosu, James Akazili, Chikezie Ifeanyi

**Affiliations:** ^1^ Department of Family Medicine and Public Health, Faculty of Medicine, University of Botswana, Gaborone, Botswana; ^2^ African Health Economics and Policy Association (AfHEA), Accra, Ghana; ^3^ Department of Community Health Services, Rady Faculty of Health Services, University of Manitoba, Winnipeg, MB, Canada; ^4^ Partnership for Economic Policy (PEP), Nairobi, Kenya; ^5^ School of Health Systems and Public Health, University of Pretoria, Pretoria, South Africa; ^6^ Okavango Research Institute, University of Botswana, Maun, Botswana; ^7^ Department of Library and Information Science, University of Botswana, Gaborone, Botswana; ^8^ Department of Community Medicine, University of Nigeria, Enugu, Nigeria; ^9^ Ethiopian Public Health Institute, Addis Ababa, Ethiopia; ^10^ Department of Public Health, Zimbabwe College of Public Health Physicians, Harare, Zimbabwe; ^11^ Department of Economics and Finance, University of the Free State, Bloemfontein, South Africa; ^12^ School of Public Health, C.K. Tedam University of Technology and Applied Sciences, Navrongo, Ghana; ^13^ BCEPS, University of Bergen, Bergen, Norway; ^14^ Health Systems and Development Research Group, Veritas University Abuja, Abuja, Nigeria

**Keywords:** COVID-19, vaccines, equitable access, vaccine hesitancy, uptake, Botswana

## Abstract

**Introduction:**

Despite global market complexities, Botswana acquired about 2.6 million COVID-19 vaccine doses between March 2021 and March 2022, 76% of which were purchased while 24% were donations. Thus, the study was envisaged to aggregate evidence on the case of Botswana's COVID-19 vaccine access patterns, hesitancy, and uptake.

**Materials and methods:**

We conducted a scoping reviewof Botswana-based articles using a predetermined search strategy to search databases including Medline, CINAHL, Web of Science, PubMed, Scopus, and Google Scholar. The review included all the English-language written peer-reviewed and grey literature reporting on vaccination in Botswana, to broaden coverage in recognition of limited publications on COVID-19 vaccinartion in Botswana. Non-English articles were excluded due to limited translation resources. Due to the heterogeneity of studies, a narrative synthesis approach was used to collect, synthesize, and map the literature.

**Results:**

As of 31 December 2021, 80.6% of the Botswana national target of 1,390,856 people over 18 years had received at least one dose of a COVID-19 vaccine, while 71.9% were fully vaccinated. Various vaccine distribution channels were utilized, including public facilities and outreaches, to improve access and uptake of vaccines. COVID-19 vaccine acceptance was considered generally high (73.4% amongst adults), and found positively associated with the male gender, those with comorbidities, those with non-restrictive religious beliefs, and those aged 55–64 years who thought the vaccine was safe for use. COVID-19 vaccine delivery relied on existing Expanded Program on Immunization (EPI) structures and therefore experienced to existing EPI challenges including, lack of transport, shortage of human resources, and vaccine stock-outs.

**Conclusions:**

Under-performance of immunization programs at the district level, characterized by declining immunization coverage and inadequate outreach services, exacerbates disparities in vaccine access. Efforts to strengthen healthcare infrastructure and expand outreach services are essential for reaching populations with limited access to healthcare facilities, particularly in rural and hard-to-reach areas. Collaboration with other government entities and the private sector improved vaccine access.

## 1 Introduction

The novel SARS-COV-2 virus led to an almost unprecedented number of coronavirus disease infections and related mortalities globally since its first cases were reported in December 2019 (1). Within a few months of its discovery, the then Coronavirus disease (later named COVID-19) was declared a global pandemic, with health systems across the globe struggling with exceptionally high patient burdens, limited infection control, and laboratory testing commodities in addition to existing insufficient capacities common to most health systems (2, 3). Over 7 million COVID-19-related mortalities and about 774 million infections were reported globally as of 4th February 2024 (4). Governments all over the world implemented non-pharmacological COVID-19 interventions, such as mandatory facial mask-wearing, school closures, regular hand washing, isolation, and quarantine services as well as total lockdowns to curb the spread of the virus. Furthermore, pharmacological interventions for prevention and control were also undertaken, of high priority being COVID-19 vaccines. Over 2 billion doses of these vaccines had been administered globally as of December 2021 (5).

The COVID-19 pandemic worsened existing vaccine distribution and access inequalities, stimulating renewed discussions about expanding vaccine research and development capabilities in Africa, to build domestic vaccine manufacturing and reduce current disparities.

COVID-19 morbidity and mortality trends in Botswana, as in many other countries in Africa, were devastating. Despite early implementation of non-pharmacological preventive measures such as partial and total lockdowns, social distancing, and mandatory mask mandates, the country's healthcare system was severely strained by the disease. Inadequate healthcare resources, including healthcare personnel, hospital beds, and ventilators, posed severe difficulties, particularly during spikes in COVID-19 cases. Moreover, disparities in healthcare access and underlying comorbidities in Botswana contributed to inequalities in COVID-19 outcomes. Vulnerable populations, such as the elderly, those with comorbidities, and those living in hard-to-reach rural areas, faced higher risks of severe illness and mortality. The socio-economic implications of the pandemic, including business and school closures, employment losses, and disruptions in healthcare service delivery, further exacerbated the crisis. As of March 1, 2021, Botswana had recorded 30,727 confirmed COVID-19 cases. A cumulative total of 330, 409 confirmed cases and 2,800 COVID-19-related deaths, since the beginning of the pandemic, were reported by December 2023 (6). These figures underscore the need for comprehensive strategies to prepare for future pandemics, including measures to ensure the prompt availability, accessibility, and acceptability of relevant vaccines.

In response to the pandemic, Botswana acquired and distributed about 2.6 million vaccine doses between March 2021 and March 2022, 76% of which were purchased while 24% were donations (2). The vaccine rollout initially focused on vulnerable groups, including healthcare workers, the elderly, and those with comorbidities, before extending to the rest of the population.

COVID-19 vaccines in Botswana were consequently delivered through the national immunization program, which provides routine immunizations for other vaccine-preventable diseases (VPDS) (2). The mission of the national immunization program, the Expanded Program in Immunization (EPI), is to provide immunization delivery services that are free, safe, and of good quality to the population (7). Under the stewardship of the Child Health Division in the Ministry of Health, the EPI delivers its immunization services through a network of public health facilities (hospitals, clinics, health posts, outreach/mobile stops) and the private sector. Recently, the program has provided 12 government-funded routine vaccination antigens, with the COVID-19 vaccine as the thirteenth (13th). The delivery of scarce COVID-19 vaccines to the population in Botswana relied heavily on using existing immunization structures, stakeholder engagement and lessons learnt from previous immunization campaigns.

Although the Government of Botswana made significant efforts to increase COVID-19 vaccine coverage, several challenges persisted. Issues of COVID-19 vaccine hesitancy and low uptake, alongside other barriers to equitable access remained unresolved. Vaccine hesitancy was recognized as one of the major deterrents to achieving herd immunity and high vaccination coverages in African countries among different population groups. In a study by Myburgh et al. (8), concerns about vaccine side effect and the high pace at which vaccines were developed led to vaccine hesitancy amongst South African and Zimbabwean populations. Vaccine safety concerns and potential adverse effects also created widespread doubts, which lead to decreased vaccination rates (9). Other studies have also shown that limited vaccine supply, combined with delivery challenges in low-income countries resulted in unequal vaccination outcomes across different population groups (10, 11). Furthermore, the fear vaccine-related adverse events by communities, physical and logistical barriers experienced during crucial pandemic times were reported to have led to disparities in vaccine coverage rates across various demographic groups (12, 13). Similar concerns exist in Botswana as they do in other African countries. Therefore, this study aimed to assess access patterns, hesitancy, and uptake of COVID-19 vaccines, as well as the barriers to equitable and timely access and uptake of vaccines in Botswana, and explore how these barriers can be mitigated.

## 2 Materials and methods

We conducted a scoping review based on its ability to identify trends and gaps in an existing knowledge base to inform research, policy, and practice (14). This scoping review was conducted following the analytic framework of Arksey and O’Malley (15). Arksey and O'Malley developed a five-stage methodological framework to guide researchers in conducting scoping reviews (15). The following five-stage framework is proposed: [1] identifying the research questions; [2] searching for relevant studies; [3] selecting studies; [4] charting the data; and [5] collating, summarizing, and reporting the results (15).

### 2.1 Search strategy

A database search was conducted between the 4th and 5th of February 2024 (and updated on the 07th of June 2024), using a predetermined search strategy (Supplementary Appendix A). The search strategy used keywords combined with their synonyms using the Boolean Operator “OR”, and to sort for the literature reporting on the intersection between all the keywords, the Boolean Operator “AND” was used.

**Figure 1:**
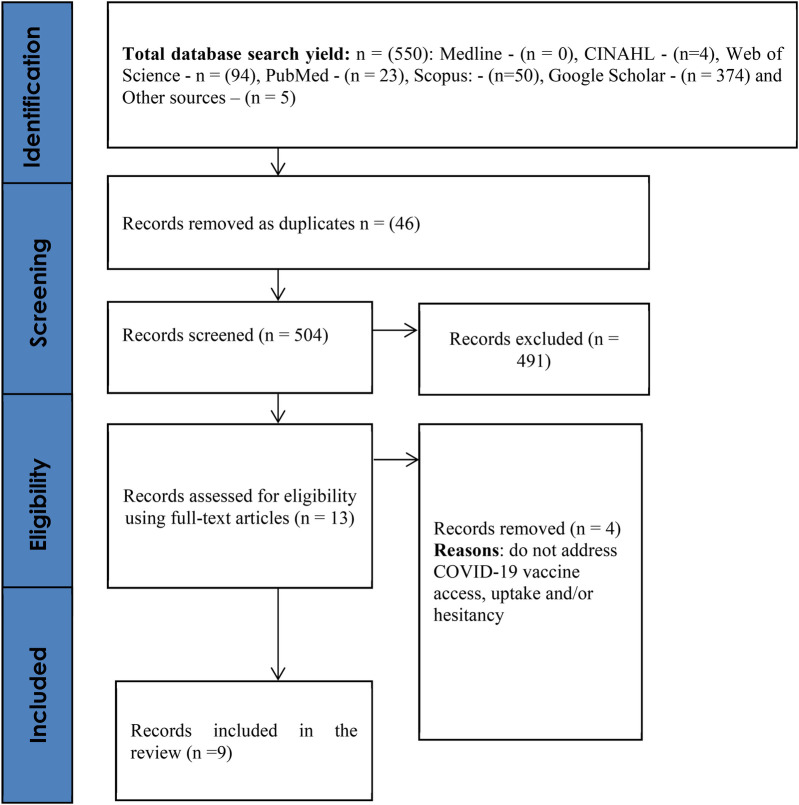
PRISMA flow diagram.

The predetermined search strategy was modified for the databases including Medline, CINAHL, Web of Science, PubMed, Scopus, and Google Scholar. The researchers conducted backward snowballing to identify relevant articles in the reference sections of the recruited studies and forward snowballing to locate relevant studies that cited the recruited studies. Moreover, all articles by authors who had two or more first/senior-author articles in the final list were also reviewed. Furthermore, the researchers looked for relevant articles in the reference section of the relevant publications (backward snowballing) and publications that cited the relevant studies (forward snowballing) (16) to ensure that all the relevant literature was captured. The search and screening processes followed PRISMA-ScR guidelines and in total, the database searches, snowballing, and studies from other sources (e.g., colleagues) yielded 550 results,before duplicates were removed, that were further screened for relevance (Figure 1). Hand searches were performed on search engines and websites including Google Scholar, Google, Research4Life—HINARI, AJOL, African Index Medicus for grey literature, including sourcing from Ministry of Health and other government portals and websites, government offices and Non-Governmental organisations websites. Each database yielded the following: Scopus (50), Google Scholar (374), Web of Science (94), PubMed (23), CINAHL (4), Medline (0) and 5 additional records (grey literature) from other sources.

### 2.2 Inclusion/exclusion criteria

The review included all the English-language written peer-reviewed and grey literature reporting on vaccination in Botswana, to broaden coverage in recognition of limited publications on COVID-19 vaccinartion in Botswana. Non-English articles were excluded due to limited translation resources. Although the COVID-19 vaccination inspired the study's inception, the study sought literature on immunization in general, considering shared delivery structures with the COVID-19 vaccine, but filtered to human vaccines. The review used a modified PICOS framework which included; Population as individuals and vaccination systems within Botswana, the Intervention as COVID-19 vaccination policies, strategies and implementation, with no Comparator and Outcomes as COVID-19 vaccine access, distrinution and hesitancy. The Study types included published journal articles and grey literature including quantitative, qualitative, and mixed-method studies.

Time limiters or filters were not imposed on the search. The screening of the results involved using the above inclusion and exclusion criteria (English-language written peer-reviewed and grey literature reporting on vaccination in Botswana) and reviewing titles, abstracts, and full texts. The screening, extraction and validation of the literature was conducted independently by the two reviewers (J. T.T. and K.K.) and they met to resolve conflicting views. A total of nine (*n* = 9) studies were included in the review (Figure 1 including five (*n* = 5) from other sources including portals, websites, and government offices.

### 2.3 Data extraction

A standardized data extraction sheet in Microsoft Excel was used to collate and categorize the data into themes, summarizing studies and reports. The following headings were used to extract detailed information for the included studies: authors, title and year of publication; study setting, i.e., the country as shown in Table 1. The methodology section comprises study characteristics, including study design, study participants, and sample size.

**Table 1:** Characteristics of reviewed studies and reports.

Author	Title	Country	Study participants	Year of publication	Sample size	Study design	Hesitancy/ Acceptance		Patterns in access and uptake	Barriers to equitable and timely access	Access improvement for vulnerable groups
							Factors	(%)			
Ministry of Health & Wellness	The Botswana Expanded Program on Immunization Multi-Year Plan 2018–2022	Botswana		2016					Vaccination incorporated into the school health programme.	Weak surveillance Poor data management Low vaccine coverages. Insufficient outreach services - -No strategy for hard to reach and un- reached populations. - -Absence of EPI policy document and guidelines at district level No Community based surveillance system - -No maintenance and replacement plan for dysfunctional equipment. - -Some facilities still using domestic refrigerators for vaccine storage. Vaccine stock-outs at district and facility levels. -Lack of stock records at facility level. -Vaccine wastage not monitored at all levels. -Vaccines target population unknown Poor vaccines management at District and facility level. -Inadequate transport. -No qualified District Cold Chain Technicians. -Lack of communication strategy -Insufficient social mobilisation -Poor community engagement at DHMTs Inappropriate management of human resources. -Human resource shortage. -Inadequate supervision at district level. -High staff transfers/rotations.	Improve awareness creation, demand generation, attitude change and community participation. Collaboration with community, religious, political leaders, teachers, women's groups, traditional. o Health workers refresher trainings. Public- private partnerships for resource mobilization. o DHMTs to develop internal annual plans & budget should - - Develop comprehensive EPI guidelines provision of transport for outreach services. o Develop written periodic preventive maintenance plans for buildings and cold chain equipment. - -Regular supportive visits to all vaccine stores - -maintain stock records for vaccines - -Develop standard operating procedures for vaccine supply chain. o strengthen surveillance network - -Regular supervisory visits from national to district and from district to health Facility o Linkage of Community Surveillance System to the formal surveillance structures. Conduct data quality review. o Analyze & use data for action - -Map hard to reach communities and resistant groups, create dialogue, and intervene. - -Develop communication strategy
Ministry of Health	Botswana Comprehensive Immunization Programme Review	Botswana	Unclear	2022		Mixed methods		Acceptance Good COVID-19 Vaccine acceptance	COVID-19 vaccine coverage at 68% overall and 70% for the high-risk groups. Phased vaccine rollout targeting 55+ year, health workers and persons with co-morbidities, persons 30+, then 18+ until targeting children aged 5+ years		Resource sharing between routine immunization & COVID-19 vaccinations Additional cold chain equipment Public-private partnerships Efficient mobilization of stakeholders Document the experience roll-out of COVID-19 vaccines in Botswana. Integration of COVID-19 vaccination into EPI
Handy et al	The impact of access to immunization information on vaccine acceptance in three countries	Botswana, Dominican Republic & Greece	Providers & Caregivers of under 5 s	2017	96 providers and 153 caregivers	Qualitative	Acceptance vaccine information from health care providers			Inadequate health providers’ knowledge, skills, or compassion	
Tlale et al.	Acceptance rate and risk perception towards the COVID-19 vaccine in Botswana	Botswana	Adults	2022	5,300	Survey	Acceptance Males Aged 55–64 Primary school education comorbidities. Non-restrictive religious beliefs Ages 55–64 believe the vaccine is safe. Willing to wear mask Trusted the government sources of information radio as preferred mode of communication	Acceptance 73.4%			
Ministry of Health	Estimation of COVID-19 delivery costs in Botswana	Botswana	DHMT-level stakeholders	2023	19 KIs and records	Mixed methods (Records review and Qualitative)			Vaccine delivery mainly facility-based, with some delivery through outreach, drive-through sites (in the two cities only) Facilities provided outreach to farms and schools	High costs of new vaccine provision to an entire population in pandemic setting compared to routine childhood vaccination Need for surge human resources	Additional Financial investments beyond what immunization programs required in pandemic. Public-private partnerships and collaboration
Wariri et al	COVID-19 vaccination trajectory and the speed needed to reach at least 70% population coverage in 52 African countries: a modelling study	52 countries		2023		Modelling			Botswana started COVID-19 vaccine rollout in March 2021 with donations from India. (30 000) vaccines) and a purchase of Sinovac from China in September 2021 (404,494 doses)		
WHO Botswana	Biennial Report 2020–2021	Botswana		2022					By 31 December 2021, 80.6% of national target received at least one dose of vaccine and 71.9% were fully vaccinated		Design of national vaccine deployment plan Support for MOH to carry out stock inventory and monthly consumption rates of all COVID-19 commodities. Procurement of COVID-19 commodities through the WHO portal platform WHO technical support for cold chain, vaccine management, capacity building of health workers on COVID-19 Vaccine Management.
WHO Arica	WHO country cooperation strategy 2014–2020, Botswana			2014						Hard to reach areas as a result of geographical limitations. Cultural norms, values and religious beliefs	Implementation of the Global Vaccine Action Plan (2011–2020) promote equitable access to quality vaccines & M&E Perform post vaccine introductory evaluation. Effective vaccine management assessment Data quality assessment Immunization coverage surveys
Mathur et al	Insights to COVID-19 vaccine delivery: Results from a survey of 27 countries	27 countries	MSH or UNICEF technical experts	2023	21 participants from 15 countries and 30 participants from 27 countries	Longitudinal Surveys			Few of countries targeted PLHIV, excluding Botswana with high HIV prevalence		Increase in cold chain capacity at country level. Targeting of special groups such as people with comorbidities, prison populations, pregnant women, refugees/IDPs, elderly, students/youth (12–17), essential workers i.e., soldiers, teachers, security personnel, legislature, judiciary, executive staff

### 2.4 Quality assurance

The reviewers together extracted the data from three (*n* = 3) articles for rapport and continued to extract independently. The reviewers met to perform a quality check, ensuring consistent extraction of data, completeness of data, and finalizing the screening for relevance.

### 2.5 Data synthesis and analysis

Due to the heterogeneity of studies, a narrative synthesis approach was used to collect, synthesize, and map the literature (17). The following predetermined categories were used to guide the analysis: [1] patterns in access and uptake of the COVID-19 vaccines in Botswana [2] barriers to equitable and timely access and uptake of COVID-19 vaccines; and [3] what is needed to improve these barriers for disadvantaged and vulnerable groups. The data extraction form (review matrix) was analyzed thematically (18), and by grouping data into categories and sub-categories (themes and subthemes), also, repeatedly comparing the sub-categories to establish relationships between them.

### 2.6 Ethical considerations

This study synthesized secondary data from publicly available official reports and peer-reviewed articles, and provided a detailed process followed for selecting the reviewed articles, including the extracted data. None of the data that was extracted and synthesized contained any confidential or sensitive information about study participants; therefore, ethical clearance was not required.

## 3 Results

### 3.1 Article characteristics

Five (*n* = 5) of the reviewed papers were published journal articles, two (*n* = 2) of which focused on Botswana only, while others investigated Botswana and other countries. The rest of the papers (*n* = 5) were official reports authored by the Ministry of Health and the WHO. Three (*n* = 3) articles used only quantitative survey methods, one (*n* = 1) qualitative method, and one (*n* = 1) quantitative modeling study, while the rest used mixed methods study design.

As shown in Table 2, COVID-19 vaccine access, hesitancy and delivery were influenced by equity dimensions such as place of residence, education level, gender, culture and religious beliefs.

**Table 2:** Summary table of themes, findings, equity dimensions and policy implications

Theme	Selected findings	PROGRESS-plus dimensions	Improvement strategies
Vaccine access	Hard to reach areas as a result of geographical limitations. Facility-based, with some delivery through outreach, drive-through sites (in the two cities only)	Place (Rural-Urban)	Increase in cold chain capacity at country level. Mobile clinics for remote areas Public- private partnerships for resource mobilization
Hesitancy and vaccine acceptance	73.4% national acceptance Males, those aged 55–64 and those with primary school education more likely to accept vaccines Prohibitive Cultural norms, values and religious beliefs	Education Gender Culture Religion Ethnicity	Gender sensitive messaging to address specific gender concerns Campaigns tailored for high literacy populations Collaborative vaccine campaigns with community, religious, political leaders, teachers, women's groups and traditional leaders
Vaccine delivery barriers	Vaccine stock-outs at district and facility levels	Place	Maintain stock records for vaccines develop standard operating procedures for vaccine supply chain at district level

### 3.2 Patterns in access and uptake of the COVID-19 vaccines

#### 3.2.1 Vaccine availability and access

Available studies and reports indicate that Botswana has a mature immunization program, which has enabled relatively good vaccine coverage in the country. However, the recent comprehensive review of the EPI reveals various challenges that are significantly weakening the EPI program, resulting in a decline in vaccines reaching targeted individuals and communities. Provided through static and mobile facilities, the EPI played a fundamental role in supporting the provision of COVID-19 vaccines.

According to an EPI review (7), the COVID-19 vaccine roll-out in Botswana was done in phases, with the first phase targeting persons aged 55 years and above, health workers, and persons with co-morbidities, the second phase including persons 30 years of age and above; the third phase including those of age 18 years and above and finally the fourth phase, targeting children aged 5 years and above. As of 31 December 2021, 80.6% of the Botswana national target of 1,390,856 people aged 18 years and above had received at least one dose of the vaccine, while 71.9% were fully vaccinated (13–15, 17, 19). The first batch of vaccines was reported to have been only 30000 donated by India in March 2021, until the next doses were purchased from China six months later (20). In another study (2), COVID-19 vaccine delivery in the country was through health facilities, with some delivery through outreach services, drive-through sites (in cities), and in schools, from 1st March 2021 to 31st March 2022. Health facilities also conducted outreach services to farms and schools in their catchment areas. However, the used vaccine doses may have frequently been reported under health facilities, not reflecting the outreach areas (2).

#### 3.2.2 Demographics and acceptance

A study by Tlale et al. (21) reported a national adult COVID-19 acceptance rate of 73.4%, with males having higher odds of accepting the COVID-19 vaccine compared to females. Those with primary school education levels had higher odds of accepting the COVID-19 vaccine compared to those with post-graduate training, and those with non-restrictive religious and cultural beliefs had higher odds of vaccine acceptance. In the same study, Individuals aged 55–64 years were also more likely to accept the COVID-19 vaccine compared to those aged 65 years and above. This was partly because Individuals aged 55–64 believed the vaccine was safe to use (21) compared to those aged 65 and above, as reported in the paper.

#### 3.2.3 Perception of disease severity and presence of co-morbidities

According to Tlale et al. (21), those participants who had comorbidities or diseases other than COVID-19 had higher odds of accepting the COVID-19 vaccine.

#### 3.2.4 Sources of health information

The population in the study by Tlale et al. (21) described COVID-19 vaccine information from healthcare providers as positively supporting COVID-19 vaccine acceptance. Government, healthcare workers, and the World Health Organization (WHO) were the most trusted sources of COVID-19 vaccine information (21, 22), although healthcare providers were described as lacking in knowledge, skills, or compassion for clients in another study (22). Radio was the most preferred mode of communication for COVID-19 vaccine-related messages (21).

#### 3.2.5 Health-seeking behavior and vaccine safety perception

Tlale et al. (21) reported that among the study participants, those who were willing to wear facial masks, as a COVID-19 prevention measure, also believed that COVID-19 vaccines were safe to use.

### 3.3 Barriers to timely access and uptake of COVID-19 vaccines

#### 3.3.1 Weak vaccine surveillance systems and poor monitoring & evaluation

In a situational analysis for EPI planning (23), the surveillance system for Vaccine Preventable Diseases (VPD) was found to be weak in active search for cases and non-vaccinated populations. This resulted in findings of reductions in surveillance indicators. Similarly, the system was found to perform poorly on regular sensitization to healthcare workers and traditional/spiritual healers on vaccine-related information, forfeiting potential influence on the population as a benefit (23).

A community-based surveillance system was reportedly absent from the program. Moreover, vaccine-related data collection, transmission/reporting, and analysis were found to be insufficient (23), therefore, there was minimal evidence of data-driven action. The District Health Management Teams (DHMTs) did not conduct any data analysis to inform local decision-making.

#### 3.3.2 Under-performance of immunzation program at the district level

Districts in Botswana were reported to have declining immunization coverage for routine vaccines with insufficient outreach service delivery (23). This may hinder vaccine delivery to populations who are unable to access facility-based services due to financial, socio-cultural, or geographical barriers. Although another study (21) reported that participants whose religious beliefs did not hinder vaccination were more likely to take the vaccine, other authors (24) also indicated the presence of hard-to-reach populations for reasons of cultural norms, values, and religious beliefs and because of geographical/physical limitations in the country. This is compounded by the findings of the EPI situational analysis (23) which found that there exists no targeted strategy for hard-to-reach and unreached populations.

The immunization program is also reported to lack a communication strategy, leading to inadequate social mobilization and poor engagement of local community structures. DHMTs did not have the EPI policy document and guidelines while they were program implementers. Furthermore, some districts did not know the program's target populations (23).

#### 3.3.3 Poor infrastructure

Concerning cold chain management, the EPI review, and multiyear plan (2, 23) reported inadequacies in refrigerated vaccine transportation, the use of domestic refrigerators for storing vaccines at the district level (23), and lack of maintenance and replacement plans for faulty cold chain equipment. However, in a study of 27 countries (25) the authors reported an increase in cold chain capacity in all countries, (including Botswana), by May 2022. Yet, 52% of the countries still reported inadequate capacity at regional levels (25).

#### 3.3.4 Inadequately trained vaccine delivery personnel

There were concerns about the limited knowledge, skills, and compassion of vaccine delivery healthcare providers at the district level (24), with no established posts of cold chain technicians in the districts (23). Additionally, studies found human resource shortages, poor management of human resources, and high staff transfers and redeployments as factors contributing to poor vaccine delivery, coverage, and uptake (2, 23). According to a COVID-19 vaccine delivery cost study (7), the Botswana Ministry of Health and the DHMTs hired at least 3,352 additional staff (surge capacity) to support COVID-19 vaccine delivery efforts, However, numbers were still not sufficient due to chronic staff shortages as alluded to in prior studies (2, 23).

#### 3.3.5 Poor vaccine management

According to some studies (2, 23), there were indications of frequent vaccine stock-outs both at DHMT and health facility levels, while vaccine stock records were also lacking in facilities. Moreover, vaccine wastage was not monitored or recorded at any level of the health system.

#### 3.3.6 Limited COVID-19 vaccine information

Most participants reported a need for more information, a scenario that may result in vaccine hesitancy, most likely through the radio or television, which were described as the preferred modes of communication regarding COVID-19 vaccine messaging (21). Additionally, participants in the same study (21) singled out the government and the WHO as reliable sources of such health-related information.

### 3.4 Improvement of COVID-19 vaccination access by vulnerable groups

Based on the presence of the Botswana Integrated Disease Surveillance and Response system that incorporates EPI; AFP, Measles, and Neonatal Tetanus within the framework of the national integrated disease surveillance (23), and COVID-19 vaccine delivery through the EPI program channels, the Botswana Comprehensive Immunization Programme Review recommended that the COVID-19 vaccination process be fully integrated into the EPI to take advantage of the well-established program (2). Furthermore, all immunizations have been incorporated into the existing school health program (23) and this may be beneficial in improving COVID-19 vaccine access. There was also an identified need for strengthening the surveillance of VPDs throughout all health system levels for improved program outcomes (23).

In a study of 27 countries including Botswana (25), COVID-19 vaccine plans in 29% of the countries, excluding Botswana, focused on People living with HIV. According to this study, Botswana did not report targeting other vulnerable groups such as prisoners, pregnant women, refugees, and any others as it was noted for other countries in the study.

However, the Botswana Comprehensive Immunization Programme Review reported that the COVID-19 vaccination rollout was phased out, with the first phase prioritizing the elderly, those with comorbidities, and healthcare workers. This was based on these groups’ higher risk of severe disease and mortality, as well as high occupational exposure and avoidance of work absenteeism due to COVID-19 illness and convalescence, in the case of the health workforce, who were already often reported to be of short supply (2, 23).

Another WHO report (24) recommended that the country intensify efforts toward improving vaccine coverage in hard-to-reach communities. According to a vaccine costs study, the Ministry of Health also collaborated with other government entities like the Ministry of Defense and Security for COVID-19 vaccine safe transportation and wide distribution and the private sector to improve vaccine access for unreached populations. Additionally, private Safari camps provided transport to deliver vaccines and health care providers to hard-to-reach areas as some of the vaccine inequity mitigation strategies (7).

#### 3.4.1 Advancement of cold chain and vaccine stock management at all health system levels

The MOH and Central Medical Store (CMS) conducted stock inventories and monthly consumption rates of all COVID-19 supplies. The WHO also supported the strengthening of cold chain and vaccine management and training of healthcare workers on COVID-19 Vaccine management (19) to improve COVID-19 vaccine access, uptake, and vaccination outcomes.

Moreover, the Botswana Expanded Program On Immunisation Multi-Year Plan 2018–2022 suggests that vaccine stock records and standard operating procedures for vaccine delivery should be developed and made accessible to all facility healthcare providers for reference, to support effective, timely, and equitable distributions of vaccines in Botswana (23).

## 4 Discussion

The findings of this study shed light on the diverse factors that influence access to and uptake of COVID-19 vaccines in Botswana. Understanding these patterns and barriers is crucial for informing effective strategies to enhance vaccination access and uptake, and address disparities in vaccine coverage.

The influence of disease severity perception and co-morbidities on vaccine acceptance is evident, with individuals perceiving diseases as severe or having underlying health conditions showing a higher inclination towards COVID-19 vaccination. These findings differ from those reported in the literature from other settings (26, 27), where those with comorbidities were more hesitant towards the COVID-19 vaccines. This underscores the importance of tailored communication strategies to dispel misinformation and increase awareness of vaccination benefits, particularly among high-risk populations such as those with comorbidities.

The reliance on trusted sources of health information, such as government sources, healthcare workers, and the WHO, emphasizes the significance of effective communication channels in shaping vaccine acceptance. The radio as a preferred communication medium highlights the need for targeted messaging to reach diverse populations, especially those in remote areas with limited access to other forms of media. This is consistent with a Sub-Saharan African study (28) where those who received pandemic information through radio were less likely to be COVID-19 vaccine-hesitant.

Several barriers impede access and uptake of COVID-19 vaccines in Botswana. Demographic factors such as education level, geographical distance, and involvement in decision-making processes influence vaccine acceptance. Males in this study were also found to be more likely to accept COVID-19 vaccines compared to females.

In one study (29) participants with more than a primary school education were less likely to get the HPV vaccine for their daughters. Those involved in the decision-making process to get the HPV vaccine for their daughters were more willing. According to the same study (29), participants who were more likely to get their daughters vaccinated were those who thought it would be easy to access vaccines. Perception of difficulties in accessing vaccines, poor engagement regarding eligibility for vaccines, and long distances to vaccine sites were found to encourage vaccine hesitancy in a study in rural South Africa (30).

Furthermore, a more recent study comparing hesitancy and resistance rates of Sub-Saharan Africa local and diaspora-based citizens also found that those with lower educational status were more likely to accept COVID-19 vaccines compared to their more educated counterparts (31). These findings were also corroborated by an extensive online survey conducted in the US (32). The finding can be attributed to a combination of social and psychological factors. The less educated tend to follow recommendations from government and health authorities without as much skepticism (33). They might feel more vulnerable to COVID-19 due to their job types, such as frontline or essential work, making them more inclined to get vaccinated for protection. The more educated individuals on the other hand may have greater access to a wide range of information, including both credible sources and misinformation, compared to the less educated (34), which may fuel their hesitancy towards COVID-19 vaccines.

The finding that males were more likely to accept the COVID-19 vaccine than females is supported by a multi-country study from Sub-Saharan Africa (35) and a meta-analysis of cross-sectional studies (36). Some possible explanations for this include the concerns of women about the adverse effect of the vaccines on fertility (36), the perceived severity of COVID-19 infection and disease by men, compared to the safety of the vaccine (37), and the economic pressures faced by men to protect their families and avoid work absenteeism due to COVID-19 infection (38).

Findings from across Southern Africa Tan et al. (39) and Harapan et al. (40) generally suggest that most populations were hesitant about COVID-19 vaccines due to fears of adverse effects of the vaccine and concerns about efficacy. These barriers can be overcome by engaging community leaders and influencers to discuss vaccine safety and misinformation. Local champions can help build a bridge between health care authorities and the community to develop a more effective public health response to increase vaccine acceptance (41, 42).

The socio-economic situation in South Africa and the history of the country have played a significant role in shaping attitudes toward vaccines. Studies have shown that vaccine acceptance is influenced by demographic factors such as race and education, and that higher educational attainment does not necessarily result in high vaccine acceptance (12, 43), corroborating the findings form Botswana. Additionally, peoples’ views on the safety and efficacy of vaccines and reluctance to get vaccinated was fuel by misinformation on social media platforms (44, 45).

Still experience differences in how aware people are of COVID-19 vaccines and how willing they are to get vaccinated. The willingness to be vaccinated in Zimbabwe and Zambia was reported to be related to how people perceived their risk of getting COVID-19 and the credibility of the sources of information (15, 46). A study in Zambia also indicated that social factors and influencer dynamics in the community were crucial in determining vaccination acceptance (47). Similarly, in Malawi, cultural beliefs and practices within the community either facilitated or hindered the vaccination efforts as was found in Botswana, and therefore, highlighting the need for local contexts considerations in the design and dissemination of health messages (41).

Geographical/physical inaccessibility to vaccine reported in this study for hard reach areas in Botswana, was also highlighted as challenge in Namibia, especially among rural populations with limited healthcare facilities, further exacerbating barriers to COVID-19 vaccine uptake (43).

## 5 Strength and limitations

This study faced challenges due to the lack of published and grey literature specifically focusing on COVID-19 vaccination in Botswana. Existing evidence mainly centered around the Expanded Program on Immunization (EPI) and other vaccine-preventable diseases. This, therefore, limited the analysis of data on COVID-19 vaccine uptake, access, hesitancy, and detailed qualitative insights into access barriers.

The demographic representation in the reviewed articles did not fully mirror Botswana's diverse population. Most studies focused on groups like healthcare workers, other officials and technical experts. Furthermore, limited information was available regarding the experiences of communities such as refugees, prisoners, and individuals with disabilities. This gap may have led to some misjudgment of the challenges encountered by these vulnerable groups. Regarding study methodologies, both cross-sectional and longitudinal studies were part of the review. However, a prevalence of cross-sectional studies restricted a comprehensive analysis of long-term trends and impacts related to identified barriers and interventions. Despite these constraints, the review lays a foundation for understanding critical barriers and prospects for improving equitable access to COVID-19 vaccines in Botswana.

## 6 Further research

Although documentation of experiences through the vaccine delivery process was recommended, there is a paucity of related published data. This review fills this gap by summarizing the available data from EPI reports, external and internal publications, and other grey literature. Future studies should focus on qualitative work to understand the opportunities for public-private partnerships, in-country vaccine development, and service delivery improvement as well as quantitative community surveys to elicit barriers to vaccine access at the personal level and assess equity in vaccine distribution. This would enable a deeper understanding of the evolving challenges and the effectiveness of interventions in improving equitable vaccine access and uptake in Botswana. Additionally, qualitative studies that give voice to the experiences of diverse, hard-to-reach, and marginalized populations would be valuable in informing more targeted and inclusive policies and programs.

The above call for comprehensive communication and education strategies, tailored to the needs and preferences of diverse population groups to address vaccine hesitancy and increase acceptance rates.

Weaknesses in vaccine surveillance systems pose significant challenges to effective vaccine distribution. Inadequate data collection and analysis hinder the identification of vaccination gaps and the implementation of targeted interventions. Strengthening surveillance systems and enhancing data-driven decision-making processes is crucial for improving vaccine coverage and monitoring uptake.

Under-performance of immunization programs at the district level, characterized by declining immunization coverage and inadequate outreach services, exacerbates disparities in vaccine access. Efforts to strengthen healthcare infrastructure and expand outreach services are essential for reaching populations with limited access to healthcare facilities, particularly in rural and hard-to-reach areas.

## 7 Conclusion

The review suggests that despite the seemingly obvious challenges, settings with limited vaccine supply such as Botswana still experience positive vaccination acceptance and uptake. Potential barriers to vaccine access and uptake such as gender, culture, religion, poor infrastructure, and lack of resources such as skilled personnel, refrigerated transportation, and storage are also highlighted. Nonetheless, there is potential for improving access and uptake of new vaccines through their integration into existing public health programs for coordination and resource sharing, provided challenges in existing programs are deliberately addressed. The utilization of multisectoral partnerships by vaccine programs can minimize the effect of vaccine access and uptake barriers.

## Data Availability

The original contributions presented in the study are included in the article/Supplementary Material, further inquiries can be directed to the corresponding author.
