# Supplementary material for: Equitable access to COVID-19 vaccines in Botswana: a scoping review
Source: Front Health Serv. 2025 Jul 29;5:1609089. doi: 10.3389/frhs.2025.1609089 (PMC12339540; doi:10.3389/frhs.2025.1609089)
Supplement: Supplementary file 1 [file Datasheet1.docx]

**APPENDIX A**

**STUDY TITLE: VACCINE EQUITY AND HESITANCY IN BOTSWANA: A SCOPE REVIEW**

**Search strategy**

**Google Scholar**

**Number of results**: 385

**Date of search**: 4^th^ February 2024 (**updated on the 07^th^ June 2024**)

Vaccination OR vaccines OR immunization OR “Immunization, Active” OR “Active Immunization” OR “Active Immunizations” OR “Immunizations, Active” AND “equitable access” OR “vaccine equity” OR “equity in vaccine uptake” OR “equity in vaccine” OR “vaccine access” OR “vaccine distribution” OR “vaccine allocation” OR “vaccine administration” OR “vaccine acceptance” OR “global distribution of Vaccines” OR “vaccine rollout” OR “vaccine rollouts” OR “vaccine Hesitancy” OR hesitance OR uncertainty OR “unwillingness” OR “marginalized communities” OR “ethnic minorities groups” OR “social inequalities” OR “marginalized and forgotten populations” OR “marginalized populations” OR Patterns OR intake OR uptake OR use OR access OR barriers OR hesitance OR obstacles OR equitable OR equity OR disfavor OR dislike OR approach OR equitable OR fairness OR timely OR “up-to-date” OR disadvantaged OR underprivileged OR “vulnerable groups” OR marginalized AND Botswana OR Batswana OR motswana

**PubMed**

**Number of results**: 23

**Date of search**: 4^th^ February 2024 (**updated on the 07^th^ June 2024**)

Vaccination[Mesh] OR vaccines[tiab] OR immunization[tiab] OR “Immunization, Active”[tiab] OR “Active Immunization”[tiab] OR “Active Immunizations”[tiab] OR “Immunizations, Active”[tiab] AND “equitable access”[tiab] OR “vaccine equity”[tiab] OR “equity in vaccine uptake”[tiab] OR “equity in vaccine”[tiab] OR “vaccine access”[tiab] OR “vaccine distribution”[tiab] OR “vaccine allocation”[tiab] OR “vaccine administration”[tiab] OR “vaccine acceptance”[tiab] OR “global distribution of Vaccines”[tiab] OR “vaccine rollout”[tiab] OR “vaccine rollouts”[tiab] OR “vaccine Hesitancy”[tiab] OR hesitance[tiab] OR uncertainty[tiab] OR “unwillingness”[tiab] OR “marginalized communities”[tiab] OR “ethnic minorities groups”[tiab] OR “social inequalities”[tiab] OR “marginalized and forgotten populations”[tiab] OR “marginalized populations”[tiab] OR Patterns[tiab] OR intake[tiab] OR uptake[tiab] OR use[tiab] OR access[tiab] OR barriers[tiab] OR hesitance[tiab] OR obstacles[tiab] OR equitable[tiab] OR equity[tiab] OR disfavor[tiab] OR dislike[tiab] OR approach[tiab] OR equitable[tiab] OR fairness[tiab] OR timely[tiab] OR “up-to-date”[tiab] OR disadvantaged[tiab] OR underprivileged[tiab] OR “vulnerable groups”[tiab] OR marginalized[tiab] AND Botswana[tiab] OR Batswana[tiab] OR motswana[tiab]

| **Search number** | **Query** | **Filters** | **Search Details** | **Results** | **Time** |
| --- | --- | --- | --- | --- | --- |
| 4 | ((Vaccination[Mesh] OR vaccines[tiab] OR immunization[tiab] OR "Immunization, Active"[tiab] OR "Active Immunization"[tiab] OR "Active Immunizations"[tiab] OR "Immunizations, Active"[tiab] AND (1000/1/1:2024/2/4[pdat])) AND ("equitable access"[tiab] OR "vaccine equity"[tiab] OR "equity in vaccine uptake"[tiab] OR "equity in vaccine"[tiab] OR "vaccine access"[tiab] OR "vaccine distribution"[tiab] OR "vaccine allocation"[tiab] OR "vaccine administration"[tiab] OR "vaccine acceptance"[tiab] OR "global distribution of Vaccines"[tiab] OR "vaccine rollout"[tiab] OR "vaccine rollouts"[tiab] OR "vaccine Hesitancy"[tiab] OR hesitance[tiab] OR uncertainty[tiab] OR "unwillingness"[tiab] OR "marginalized communities"[tiab] OR "ethnic minorities groups"[tiab] OR "social inequalities"[tiab] OR "marginalized and forgotten populations"[tiab] OR "marginalized populations"[tiab] OR Patterns[tiab] OR intake[tiab] OR uptake[tiab] OR use[tiab] OR access[tiab] OR barriers[tiab] OR hesitance[tiab] OR obstacles[tiab] OR equitable[tiab] OR equity[tiab] OR disfavor[tiab] OR dislike[tiab] OR approach[tiab] OR equitable[tiab] OR fairness[tiab] OR timely[tiab] OR "up-to-date"[tiab] OR disadvantaged[tiab] OR underprivileged[tiab] OR "vulnerable groups"[tiab] OR marginalized[tiab] AND (1000/1/1:2024/2/4[pdat]))) AND (Botswana[tiab] OR Batswana[tiab] OR motswana[tiab] AND (1000/1/1:2024/2/4[pdat])) | from 1000/1/1 - 2024/2/4 | (("vaccination"[MeSH Terms] OR "vaccines"[Title/Abstract] OR "immunization"[Title/Abstract] OR "immunization active"[Title/Abstract] OR "Active Immunization"[Title/Abstract] OR "Active Immunizations"[Title/Abstract] OR "immunizations active"[Title/Abstract]) AND 1000/01/01:2024/02/04[Date - Publication] AND (("equitable access"[Title/Abstract] OR "vaccine equity"[Title/Abstract] OR "vaccine access"[Title/Abstract] OR "vaccine distribution"[Title/Abstract] OR "vaccine allocation"[Title/Abstract] OR "vaccine administration"[Title/Abstract] OR "vaccine acceptance"[Title/Abstract] OR "vaccine rollout"[Title/Abstract] OR "vaccine rollouts"[Title/Abstract] OR "vaccine Hesitancy"[Title/Abstract] OR "hesitance"[Title/Abstract] OR "uncertainty"[Title/Abstract] OR "unwillingness"[Title/Abstract] OR "marginalized communities"[Title/Abstract] OR "social inequalities"[Title/Abstract] OR "marginalized populations"[Title/Abstract] OR "Patterns"[Title/Abstract] OR "intake"[Title/Abstract] OR "uptake"[Title/Abstract] OR "use"[Title/Abstract] OR "access"[Title/Abstract] OR "barriers"[Title/Abstract] OR "hesitance"[Title/Abstract] OR "obstacles"[Title/Abstract] OR "equitable"[Title/Abstract] OR "equity"[Title/Abstract] OR "disfavor"[Title/Abstract] OR "dislike"[Title/Abstract] OR "approach"[Title/Abstract] OR "equitable"[Title/Abstract] OR "fairness"[Title/Abstract] OR "timely"[Title/Abstract] OR "up-to-date"[Title/Abstract] OR "disadvantaged"[Title/Abstract] OR "underprivileged"[Title/Abstract] OR "vulnerable groups"[Title/Abstract] OR "marginalized"[Title/Abstract]) AND 1000/01/01:2024/02/04[Date - Publication]) AND (("Botswana"[Title/Abstract] OR "Batswana"[Title/Abstract] OR "motswana"[Title/Abstract]) AND 1000/01/01:2024/02/04[Date - Publication])) AND (1000/1/1:2024/2/4[pdat]) | 23 | 07:50:11 |
| 3 | Botswana[tiab] OR Batswana[tiab] OR motswana[tiab] | from 1000/1/1 - 2024/2/4 | ("Botswana"[Title/Abstract] OR "Batswana"[Title/Abstract] OR "motswana"[Title/Abstract]) AND (1000/1/1:2024/2/4[pdat]) | 3,183 | 07:47:52 |
| 2 | "equitable access"[tiab] OR "vaccine equity"[tiab] OR "equity in vaccine uptake"[tiab] OR "equity in vaccine"[tiab] OR "vaccine access"[tiab] OR "vaccine distribution"[tiab] OR "vaccine allocation"[tiab] OR "vaccine administration"[tiab] OR "vaccine acceptance"[tiab] OR "global distribution of Vaccines"[tiab] OR "vaccine rollout"[tiab] OR "vaccine rollouts"[tiab] OR "vaccine Hesitancy"[tiab] OR hesitance[tiab] OR uncertainty[tiab] OR "unwillingness"[tiab] OR "marginalized communities"[tiab] OR "ethnic minorities groups"[tiab] OR "social inequalities"[tiab] OR "marginalized and forgotten populations"[tiab] OR "marginalized populations"[tiab] OR Patterns[tiab] OR intake[tiab] OR uptake[tiab] OR use[tiab] OR access[tiab] OR barriers[tiab] OR hesitance[tiab] OR obstacles[tiab] OR equitable[tiab] OR equity[tiab] OR disfavor[tiab] OR dislike[tiab] OR approach[tiab] OR equitable[tiab] OR fairness[tiab] OR timely[tiab] OR "up-to-date"[tiab] OR disadvantaged[tiab] OR underprivileged[tiab] OR "vulnerable groups"[tiab] OR marginalized[tiab] | from 1000/1/1 - 2024/2/4 | ("equitable access"[Title/Abstract] OR "vaccine equity"[Title/Abstract] OR "vaccine access"[Title/Abstract] OR "vaccine distribution"[Title/Abstract] OR "vaccine allocation"[Title/Abstract] OR "vaccine administration"[Title/Abstract] OR "vaccine acceptance"[Title/Abstract] OR "vaccine rollout"[Title/Abstract] OR "vaccine rollouts"[Title/Abstract] OR "vaccine Hesitancy"[Title/Abstract] OR "hesitance"[Title/Abstract] OR "uncertainty"[Title/Abstract] OR "unwillingness"[Title/Abstract] OR "marginalized communities"[Title/Abstract] OR "social inequalities"[Title/Abstract] OR "marginalized populations"[Title/Abstract] OR "Patterns"[Title/Abstract] OR "intake"[Title/Abstract] OR "uptake"[Title/Abstract] OR "use"[Title/Abstract] OR "access"[Title/Abstract] OR "barriers"[Title/Abstract] OR "hesitance"[Title/Abstract] OR "obstacles"[Title/Abstract] OR "equitable"[Title/Abstract] OR "equity"[Title/Abstract] OR "disfavor"[Title/Abstract] OR "dislike"[Title/Abstract] OR "approach"[Title/Abstract] OR "equitable"[Title/Abstract] OR "fairness"[Title/Abstract] OR "timely"[Title/Abstract] OR "up-to-date"[Title/Abstract] OR "disadvantaged"[Title/Abstract] OR "underprivileged"[Title/Abstract] OR "vulnerable groups"[Title/Abstract] OR "marginalized"[Title/Abstract]) AND (1000/1/1:2024/2/4[pdat]) | 6,972,686 | 07:47:30 |
| 1 | Vaccination[Mesh] OR vaccines[tiab] OR immunization[tiab] OR "Immunization, Active"[tiab] OR "Active Immunization"[tiab] OR "Active Immunizations"[tiab] OR "Immunizations, Active"[tiab] | from 1000/1/1 - 2024/2/4 | ("vaccination"[MeSH Terms] OR "vaccines"[Title/Abstract] OR "immunization"[Title/Abstract] OR "immunization active"[Title/Abstract] OR "Active Immunization"[Title/Abstract] OR "Active Immunizations"[Title/Abstract] OR "immunizations active"[Title/Abstract]) AND (1000/1/1:2024/2/4[pdat]) | 309,053 | 07:47:04 |

**Medline - EBSCO**

**Number of results**: 0

**Date of search**: 4^th^ February 2024 (**updated on the 07^th^ June 2024**)

exp Vaccination/ OR vaccines.tw. OR immunization.tw. OR "Immunization, Active".tw. OR "Active Immunization".tw. OR "Active Immunizations".tw. OR "Immunizations, Active".tw. AND "equitable access".tw. OR "vaccine equity".tw. OR "equity in vaccine uptake".tw. OR "equity in vaccine".tw. OR "vaccine access".tw. OR "vaccine distribution".tw. OR "vaccine allocation".tw. OR "vaccine administration".tw. OR "vaccine acceptance".tw. OR "global distribution of Vaccines".tw. OR "vaccine rollout".tw. OR "vaccine rollouts".tw. OR "vaccine Hesitancy".tw. OR hesitance.tw. OR uncertainty.tw. OR unwillingness.tw. OR "marginalized communities".tw. OR "ethnic minorities groups".tw. OR "social inequalities".tw. OR "marginalized and forgotten populations".tw. OR "marginalized populations".tw. OR Patterns.tw. OR intake.tw. OR uptake.tw. OR use.tw. OR access.tw. OR barriers.tw. OR hesitance.tw. OR obstacles.tw. OR equitable.tw. OR equity.tw. OR disfavor.tw. OR dislike.tw. OR approach.tw. OR equitable.tw. OR fairness.tw. OR timely.tw. OR up-to-date.tw. OR disadvantaged.tw. OR underprivileged.tw. OR "vulnerable groups".tw. OR marginalized.tw. AND Botswana.tw. OR Batswana.tw. OR motswana.tw.

| **#** | **Query** | **Limiters/Expanders** | **Last Run Via** | **Results** |
| --- | --- | --- | --- | --- |
| S4 | S1 AND S2 AND S3 | Expanders - Apply equivalent subjects Search modes - Boolean/Phrase | Interface - EBSCOhost Research Databases Search Screen - Advanced Search Database - MEDLINE | 0 |
| S3 | "Botswana.tw. OR Batswana.tw. OR motswana.tw." | Expanders - Apply equivalent subjects Search modes - Boolean/Phrase | Interface - EBSCOhost Research Databases Search Screen - Advanced Search Database - MEDLINE | 0 |
| S2 | ""equitable access".tw. OR "vaccine equity".tw. OR "equity in vaccine uptake".tw. OR "equity in vaccine".tw. OR "vaccine access".tw. OR "vaccine distribution".tw. OR "vaccine allocation".tw. OR "vaccine administration".tw. OR "vaccine acceptance".tw. OR "global distribution of Vaccines".tw. OR "vaccine rollout".tw. OR "vaccine rollouts".tw. OR "vaccine Hesitancy".tw. OR hesitance.tw. OR uncertainty.tw. OR unwillingness.tw. OR "marginalized communities".tw. OR "ethnic minorities groups".tw. OR "social inequalities".tw. OR "marginalized and forgotten populations".tw. OR "marginalized populations".tw. OR Patterns.tw. OR intake.tw. OR uptake.tw. OR use.tw. OR access.tw. OR barriers.tw. OR hesitance.tw. OR obstacles.tw. OR equitable.tw. OR equity.tw. OR disfavor.tw. OR dislike.tw. OR approach.tw. OR equitable.tw. OR fairness.tw. OR timely.tw. OR up-to-date.tw. OR disadvantaged.tw. OR underprivileged.tw. OR "vulnerable groups".tw. OR marginalized.tw." | Expanders - Apply equivalent subjects Search modes - SmartText Searching | Interface - EBSCOhost Research Databases Search Screen - Advanced Search Database - MEDLINE | 2,096 |
| S1 | "exp Vaccination/ OR vaccines.tw. OR immunization.tw. OR "Immunization, Active".tw. OR "Active Immunization".tw. OR "Active Immunizations".tw. OR "Immunizations, Active".tw." | Expanders - Apply equivalent subjects Search modes - SmartText Searching | Interface - EBSCOhost Research Databases Search Screen - Advanced Search Database - MEDLINE | 74,936 |

**CINAHL**

**Number of results**: 4

**Date of search**: 4^th^ February 2024 (**updated on the 07^th^ June 2024**)

(MH Vaccination+) OR (TI vaccines OR AB vaccines) OR (TI immunization OR AB immunization) OR (TI "Immunization, Active" OR AB "Immunization, Active") OR (TI "Active Immunization" OR AB "Active Immunization") OR (TI "Active Immunizations" OR AB "Active Immunizations") OR (TI "Immunizations, Active" OR AB "Immunizations, Active") AND (TI "equitable access" OR AB "equitable access") OR (TI "vaccine equity" OR AB "vaccine equity") OR (TI "equity in vaccine uptake" OR AB "equity in vaccine uptake") OR (TI "equity in vaccine" OR AB "equity in vaccine") OR (TI "vaccine access" OR AB "vaccine access") OR (TI "vaccine distribution" OR AB "vaccine distribution") OR (TI "vaccine allocation" OR AB "vaccine allocation") OR (TI "vaccine administration" OR AB "vaccine administration") OR (TI "vaccine acceptance" OR AB "vaccine acceptance") OR (TI "global distribution of Vaccines" OR AB "global distribution of Vaccines") OR (TI "vaccine rollout" OR AB "vaccine rollout") OR (TI "vaccine rollouts" OR AB "vaccine rollouts") OR (TI "vaccine Hesitancy" OR AB "vaccine Hesitancy") OR (TI hesitance OR AB hesitance) OR (TI uncertainty OR AB uncertainty) OR (TI unwillingness OR AB unwillingness) OR (TI "marginalized communities" OR AB "marginalized communities") OR (TI "ethnic minorities groups" OR AB "ethnic minorities groups") OR (TI "social inequalities" OR AB "social inequalities") OR (TI "marginalized and forgotten populations" OR AB "marginalized and forgotten populations") OR (TI "marginalized populations" OR AB "marginalized populations") OR (TI Patterns OR AB Patterns) OR (TI intake OR AB intake) OR (TI uptake OR AB uptake) OR (TI use OR AB use) OR (TI access OR AB access) OR (TI barriers OR AB barriers) OR (TI hesitance OR AB hesitance) OR (TI obstacles OR AB obstacles) OR (TI equitable OR AB equitable) OR (TI equity OR AB equity) OR (TI disfavor OR AB disfavor) OR (TI dislike OR AB dislike) OR (TI approach OR AB approach) OR (TI equitable OR AB equitable) OR (TI fairness OR AB fairness) OR (TI timely OR AB timely) OR (TI up-to-date OR AB up-to-date) OR (TI disadvantaged OR AB disadvantaged) OR (TI underprivileged OR AB underprivileged) OR (TI "vulnerable groups" OR AB "vulnerable groups") OR (TI marginalized OR AB marginalized) AND (TI Botswana OR AB Botswana) OR (TI Batswana OR AB Batswana) OR (TI motswana OR AB motswana)

| **#** | **Query** | **Limiters/Expanders** | **Last Run Via** | **Results** |
| --- | --- | --- | --- | --- |
| S4 | S1 AND S2 AND S3 | Expanders - Apply equivalent subjects Search modes - Boolean/Phrase | Interface - EBSCOhost Research Databases Search Screen - Advanced Search Database - CINAHL with Full Text | 4 |
| S3 | (TI Botswana OR AB Botswana) OR (TI Batswana OR AB Batswana) OR (TI motswana OR AB motswana) | Expanders - Apply equivalent subjects Search modes - Boolean/Phrase | Interface - EBSCOhost Research Databases Search Screen - Advanced Search Database - CINAHL with Full Text | 1,119 |
| S2 | (TI "equitable access" OR AB "equitable access") OR (TI "vaccine equity" OR AB "vaccine equity") OR (TI "equity in vaccine uptake" OR AB "equity in vaccine uptake") OR (TI "equity in vaccine" OR AB "equity in vaccine") OR (TI "vaccine access" OR AB "vaccine access") OR (TI "vaccine distribution" OR AB "vaccine distribution") OR (TI "vaccine allocation" OR AB "vaccine allocation") OR (TI "vaccine administration" OR AB "vaccine administration") OR (TI "vaccine acceptance" OR AB "vaccine acceptance") OR (TI "global distribution of Vaccines" OR AB "global distribution of Vaccines") OR (TI "vaccine rollout" OR AB "vaccine rollout") OR (TI "vaccine rollouts" OR AB "vaccine rollouts") OR (TI "vaccine Hesitancy" OR AB "vaccine Hesitancy") OR (TI hesitance OR AB hesitance) OR (TI uncertainty OR AB uncertainty) OR (TI unwillingness OR AB unwillingness) OR (TI "marginalized communities" OR AB "marginalized communities") OR (TI "ethnic minorities groups" OR AB "ethnic minorities groups") OR (TI "social inequalities" OR AB "social inequalities") OR (TI "marginalized and forgotten populations" OR AB "marginalized and forgotten populations") OR (TI "marginalized populations" OR AB "marginalized populations") OR (TI Patterns OR AB Patterns) OR (TI intake OR AB intake) OR (TI uptake OR AB uptake) OR (TI use OR AB use) OR (TI access OR AB access) OR (TI barriers OR AB barriers) OR (TI hesitance OR AB hesitance) OR (TI obstacles OR AB obstacles) OR (TI equitable OR AB equitable) OR (TI equity OR AB equity) OR (TI disfavor OR AB disfavor) OR (TI dislike OR AB dislike) OR (TI approach OR AB approach) OR (TI equitable OR AB equitable) OR (TI fairness OR AB fairness) OR (TI timely OR AB timely) OR (TI up-to-date OR AB up-to-date) OR (TI disadvantaged OR AB disadvantaged) OR (TI underprivileged OR AB underprivileged) OR (TI "vulnerable groups" OR AB "vulnerable groups") OR (TI marginalized OR AB marginalized) | Expanders - Apply equivalent subjects Search modes - Boolean/Phrase | Interface - EBSCOhost Research Databases Search Screen - Advanced Search Database - CINAHL with Full Text | 1,701,015 |
| S1 | (MH Vaccination+) OR (TI vaccines OR AB vaccines) OR (TI immunization OR AB immunization) OR (TI "Immunization, Active" OR AB "Immunization, Active") OR (TI "Active Immunization" OR AB "Active Immunization") OR (TI "Active Immunizations" OR AB "Active Immunizations") OR (TI "Immunizations, Active" OR AB "Immunizations, Active") | Expanders - Apply equivalent subjects Search modes - Boolean/Phrase | Interface - EBSCOhost Research Databases Search Screen - Advanced Search Database - CINAHL with Full Text | 58,214 |

**Web of Science**

**Number of results**: 94

**Date of search**: 4^th^ February 2024 (**updated on the 07^th^ June 2024**)

Vaccination OR vaccines OR immunization OR "Immunization, Active" OR "Active Immunization" OR "Active Immunizations" OR "Immunizations, Active" AND "equitable access" OR "vaccine equity" OR "equity in vaccine uptake" OR "equity in vaccine" OR "vaccine access" OR "vaccine distribution" OR "vaccine allocation" OR "vaccine administration" OR "vaccine acceptance" OR "global distribution of Vaccines" OR "vaccine rollout" OR "vaccine rollouts" OR "vaccine Hesitancy" OR hesitance OR uncertainty OR unwillingness OR "marginalized communities" OR "ethnic minorities groups" OR "social inequalities" OR "marginalized and forgotten populations" OR "marginalized populations" OR Patterns OR intake OR uptake OR use OR access OR barriers OR hesitance OR obstacles OR equitable OR equity OR disfavor OR dislike OR approach OR equitable OR fairness OR timely OR up-to-date OR disadvantaged OR underprivileged OR "vulnerable groups" OR marginalized AND Botswana OR Batswana OR motswana

| **Entitlements** | **#** | **Search Query** | **Database** | **Results** | **Date Run** |
| --- | --- | --- | --- | --- | --- |
| - WOS.IC: 1993 to 2024 - WOS.CCR: 1985 to 2024 - WOS.SCI: 1985 to 2024 - WOS.AHCI: 1985 to 2024 - WOS.BHCI: 2005 to 2024 - WOS.BSCI: 2005 to 2024 - WOS.ESCI: 2019 to 2024 - WOS.ISTP: 1990 to 2024 - WOS.SSCI: 1985 to 2024 - WOS.ISSHP: 1990 to 2024 | 1 | TS=(Vaccination OR vaccines OR immunization OR "Immunization, Active" OR "Active Immunization" OR "Active Immunizations" OR "Immunizations, Active") | Web of Science Core Collection | 499548 | Fri Jun 07 2024 14:35:45 GMT+0200 (South Africa Standard Time) |
| - WOS.IC: 1993 to 2024 - WOS.CCR: 1985 to 2024 - WOS.SCI: 1985 to 2024 - WOS.AHCI: 1985 to 2024 - WOS.BHCI: 2005 to 2024 - WOS.BSCI: 2005 to 2024 - WOS.ESCI: 2019 to 2024 - WOS.ISTP: 1990 to 2024 - WOS.SSCI: 1985 to 2024 - WOS.ISSHP: 1990 to 2024 | 2 | TS=("equitable access" OR "vaccine equity" OR "equity in vaccine uptake" OR "equity in vaccine" OR "vaccine access" OR "vaccine distribution" OR "vaccine allocation" OR "vaccine administration" OR "vaccine acceptance" OR "global distribution of Vaccines" OR "vaccine rollout" OR "vaccine rollouts" OR "vaccine Hesitancy" OR hesitance OR uncertainty OR unwillingness OR "marginalized communities" OR "ethnic minorities groups" OR "social inequalities" OR "marginalized and forgotten populations" OR "marginalized populations" OR Patterns OR intake OR uptake OR use OR access OR barriers OR hesitance OR obstacles OR equitable OR equity OR disfavor OR dislike OR approach OR equitable OR fairness OR timely OR up-to-date OR disadvantaged OR underprivileged OR "vulnerable groups" OR marginalized) | Web of Science Core Collection | 31557555 | Fri Jun 07 2024 14:36:34 GMT+0200 (South Africa Standard Time) |
| - WOS.IC: 1993 to 2024 - WOS.CCR: 1985 to 2024 - WOS.SCI: 1985 to 2024 - WOS.AHCI: 1985 to 2024 - WOS.BHCI: 2005 to 2024 - WOS.BSCI: 2005 to 2024 - WOS.ESCI: 2019 to 2024 - WOS.ISTP: 1990 to 2024 - WOS.SSCI: 1985 to 2024 - WOS.ISSHP: 1990 to 2024 | 3 | TS=(Botswana OR Batswana OR motswana) | Web of Science Core Collection | 8622 | Fri Jun 07 2024 14:37:13 GMT+0200 (South Africa Standard Time) |
| - WOS.IC: 1993 to 2024 - WOS.CCR: 1985 to 2024 - WOS.SCI: 1985 to 2024 - WOS.AHCI: 1985 to 2024 - WOS.BHCI: 2005 to 2024 - WOS.BSCI: 2005 to 2024 - WOS.ESCI: 2019 to 2024 - WOS.ISTP: 1990 to 2024 - WOS.SSCI: 1985 to 2024 - WOS.ISSHP: 1990 to 2024 | 4 | #3 AND #2 AND #1 | Web of Science Core Collection | 94 | Fri Jun 07 2024 14:37:33 GMT+0200 (South Africa Standard Time) |

**Scopus**

**Number of results**: 50

**Date of search**: 5^th^ February 2024 (**updated on the 07^th^ June 2024**)

INDEXTERMS ( vaccination ) OR TITLE-ABS ( vaccines ) OR TITLE-ABS ( immunization ) OR TITLE-ABS ( "Immunization, Active" ) OR TITLE-ABS ( "Active Immunization" ) OR TITLE-ABS ( "Active Immunizations" ) OR TITLE-ABS ( "Immunizations, Active" ) AND TITLE-ABS ( "equitable access" ) OR TITLE-ABS ( "vaccine equity" ) OR TITLE-ABS ( "equity in vaccine uptake" ) OR TITLE-ABS ( "equity in vaccine" ) OR TITLE-ABS ( "vaccine access" ) OR TITLE-ABS ( "vaccine distribution" ) OR TITLE-ABS ( "vaccine allocation" ) OR TITLE-ABS ( "vaccine administration" ) OR TITLE-ABS ( "vaccine acceptance" ) OR TITLE-ABS ( "global distribution of Vaccines" ) OR TITLE-ABS ( "vaccine rollout" ) OR TITLE-ABS ( "vaccine rollouts" ) OR TITLE-ABS ( "vaccine Hesitancy" ) OR TITLE-ABS ( hesitance ) OR TITLE-ABS ( uncertainty ) OR TITLE-ABS ( unwillingness ) OR TITLE-ABS ( "marginalized communities" ) OR TITLE-ABS ( "ethnic minorities groups" ) OR TITLE-ABS ( "social inequalities" ) OR TITLE-ABS ( "marginalized and forgotten populations" ) OR TITLE-ABS ( "marginalized populations" ) OR TITLE-ABS ( patterns ) OR TITLE-ABS ( intake ) OR TITLE-ABS ( uptake ) OR TITLE-ABS ( use ) OR TITLE-ABS ( access ) OR TITLE-ABS ( barriers ) OR TITLE-ABS ( hesitance ) OR TITLE-ABS ( obstacles ) OR TITLE-ABS ( equitable ) OR TITLE-ABS ( equity ) OR TITLE-ABS ( disfavor ) OR TITLE-ABS ( dislike ) OR TITLE-ABS ( approach ) OR TITLE-ABS ( equitable ) OR TITLE-ABS ( fairness ) OR TITLE-ABS ( timely ) OR TITLE-ABS ( up-to-date ) OR TITLE-ABS ( disadvantaged ) OR TITLE-ABS ( underprivileged ) OR TITLE-ABS ( "vulnerable groups" ) OR TITLE-ABS ( marginalized ) AND TITLE-ABS ( botswana ) OR TITLE-ABS ( batswana ) OR TITLE-ABS ( motswana )
